# Fault Detection of T-Type Three-Level Converters with Simulation-Data Transfer Learning Strategy

**DOI:** 10.3390/s26051519

**Published:** 2026-02-28

**Authors:** Xu Huang, Jianzhong Zhang, Dan Tao, Sarvarbek Ruzimov

**Affiliations:** School of Electrical Engineering, Southeast University, Nanjing 210096, China

**Keywords:** power device failure, multilevel converters, simulation data-driven, T-type, fault detection, transfer learning

## Abstract

Accurately locating switching device faults in multilevel converters remains a challenge, particularly considering the scarcity of labeled fault data in practical industrial applications. To address this, this paper proposes a data-driven fault detection framework based on a simulation transfer learning network (STLNet). First, raw three-phase current signals are preprocessed using resampling, wavelet denoising, and normalization to generate 2D current feature images. To enrich the fault samples, a symmetry-based augmentation strategy is applied. Subsequently, a lightweight convolutional neural network is pre-trained on abundant simulation data to learn fundamental fault signatures. Finally, the designed model is transferred to the real domain by fine-tuning with a minimal amount of experimental data. Experimental validation on a T-type three-level converter platform demonstrates that the proposed STLNet achieves superior diagnostic accuracy and generalization performance compared with traditional methods, while significantly reducing the dependency on real-world fault data.

## 1. Introduction

The T-type three-level converter has emerged as a preferred topology for medium-voltage drives and grid-tied renewable energy systems. It offers a superior trade-off between conduction losses and harmonic performance compared with conventional two-level or neutral-point-clamped inverters [1,2]. These attributes are particularly critical in applications like electric vehicle traction drives and wind power converters, where high efficiency and high-quality output waveforms are mandatory [3]. Despite these advantages, the increased component count, specifically the inclusion of bidirectional switches connected to the neutral point, inherently escalates the probability of semiconductor failures. Open-circuit faults induced by thermal cycling, bond wire fatigue, or gate driver failures can lead to severe consequences. These include DC-link voltage imbalance, overstress on remaining healthy devices, and significant output current distortion, which may eventually cause catastrophic system breakdown [4]. Therefore, a robust and rapid fault diagnosis mechanism is indispensable to trigger protective measures and prevent cascading system failures [5].

Fault diagnosis methodologies generally fall into three categories: model-based, signal processing-based, and data-driven methods. Model-based approaches utilize observers or state estimation to generate residuals between predicted and measured variables [6]. Recent advancements have introduced digital twin technologies to create virtual replicas of physical systems for improved monitoring and diagnosis [7]. While theoretically rigorous, the efficacy of these methods is heavily compromised by parameter mismatches, such as inductance variation due to saturation or temperature, and non-linearities in real-world systems [8]. Furthermore, establishing an accurate analytical model for complex power electronic systems is often a laborious task. Signal processing techniques employing tools like the Fast Fourier Transform or Wavelet Transform rely on extracting specific frequency-domain signatures [9]. Melluso et al. [10] successfully extracted torque fault signals in hybrid electric powertrains through a wavelet-supported processing of residuals. Similarly, Chen et al. [11] proposed an interpretable Wavelet Kolmogorov–Arnold Convolutional LSTM network to enhance spatial-temporal feature extraction for intelligent fault diagnosis. Some recent studies have proposed using specific features like zero-crossing currents or modulation voltage differences to identify faults in T-type converters [12]. However, these methods often necessitate expert knowledge to manually set thresholds, which may not be robust against varying load conditions, variable switching frequencies, and background noise levels [13].

In recent years, data-driven approaches, particularly deep learning, have revolutionized fault diagnosis by enabling automatic feature extraction from raw signals [14]. Convolutional neural networks (CNNs) have shown exceptional capability in capturing spatial-temporal correlations in three-phase currents, often treating one-dimensional signals as two-dimensional images or time-frequency maps [15]. Despite these successes in academic settings, a critical gap between simulation and reality hinders their industrial deployment [16]. Standard deep learning models operate on the assumption that training and testing data share identical probability distributions. In practice, collecting a massive and labeled dataset of destructive faults from an operational MW-scale converter is prohibitively costly and dangerous [17]. Inducing real faults can physically damage the prototype, making the acquisition of large-scale data infeasible. Consequently, models trained on limited experimental data suffer from severe overfitting. Conversely, models trained solely on ideal simulation data fail to generalize to physical systems due to the domain shift caused by sensor noise, switching harmonics, dead-time effects, and parasitic parameters that are difficult to model perfectly [18].

To bridge this gap, transfer learning has been identified as a promising solution. This technique allows a model to learn fundamental fault signatures from a data-rich source domain and adapt these features to a data-scarce target domain [19]. By transferring the learned feature weights, the model effectively aligns the feature spaces of simulation and reality. However, existing transfer learning frameworks for power electronics often utilize heavy and pre-trained networks designed for generic image classification tasks, such as VGG-16 or ResNet [20]. These models carry millions of parameters, imposing a heavy computational burden that is incompatible with the limited storage and processing resources of embedded industrial controllers like DSPs or FPGAs [21]. Furthermore, pure data-driven augmentations like random rotation or cropping often ignore the specific topological symmetries of three-phase inverters, leading to inefficient learning and poor interpretability [22].

Addressing these limitations, this paper proposes a lightweight simulation transfer learning network (STLNet) tailored for T-type three-level converters. Unlike generic deep models, the proposed network is optimized for the specific feature space of current waveforms and explicitly addresses the data scarcity issue through a physics-informed approach [23,24,25]. The strategy leverages the abundance of low-cost simulation data to establish a robust base model, which is then fine-tuned with a minimal amount of experimental data to accommodate real-world non-linearities. Unlike prevalent transfer learning methods that depend on heavy, generic models or purely data-driven fine-tuning, the proposed approach employs a lightweight architecture designed for embedded deployment. By incorporating a physics-informed data augmentation strategy based on circuit symmetry, the proposed framework guarantees the extraction of intrinsic fault mechanisms rather than data artifacts.

The main contributions of this work are summarized as follows:

1. A sim-to-real transfer learning framework is established, which effectively aligns the feature distributions of simulation and experimental data, achieving high accuracy with minimal real-world samples.
2. A lightweight network architecture is designed, which significantly reduces the parameter count by optimizing network depth and width for current signatures, thereby facilitating embedded deployment.
3. A physics-informed data augmentation strategy is proposed, which leverages the rotational and structural symmetries of the topology to exponentially expand the effective sample size and enhance the model’s physical interpretability.

The remainder of this paper is organized as follows: Section 2 analyzes the fault characteristics. Section 3 details the proposed strategy. Section 4 describes the model construction and data processing. Section 5 presents the experimental validation, followed by the conclusion in Section 6.

## 2. Fault Characteristics

**Figure 1:**
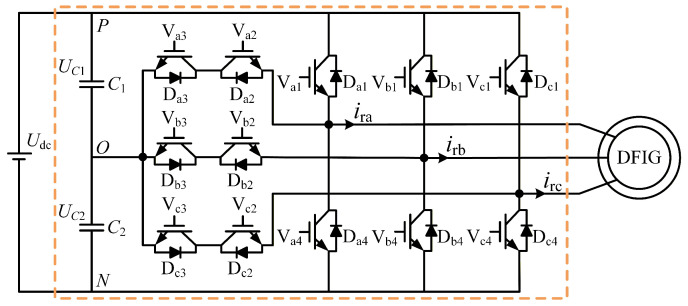
Main circuit of T-type three-level power converter for wind turbine.

Nowadays, the T-type three-level power converter is among the most widely adopted multilevel converters in renewable power industries, including wind and photovoltaic power generation. Moreover, there are more complex fault types within the T-type three-level converter due to the increased number of switching devices [26,27]. Since the machine-side converter in wind turbines has the same structure and similar control loops as the grid-side converter, the machine-side converter is employed as a case study in this work to investigate the fault mechanisms and characteristics of the power converter under diverse fault conditions, as illustrated in Figure 1.

Figure 1 shows a total of 12 IGBTs in the main circuit, where 6 IGBTs $V_{x1}$ and $V_{x4}$ (x=a,b,c) consist of the vertical arms, and the other 6 IGBTs $V_{x2}$ and $V_{x3}$ (x=a,b,c) consist of the horizontal arms of the T-type converter. The most common faults of switching devices are short circuit and open circuit. Since a short circuit in an IGBT can readily transition into an open circuit through the protection system, only IGBT open-circuit faults are focused on in the following research.

### Switching Function Model

For clarity, in the following description, the subscript *x* represents the phase index (x∈{a,b,c}), and the numerical subscripts 1 to 4 denote the specific IGBT switches within a single phase leg, as shown in Figure 1. For instance, $S_{a1}$ refers to the top switch of Phase A. Because of the symmetrical structure in the T-type three-level converter, only phase A is examined as a case study. Suppose $S_{a1}$, $S_{a2}$, $S_{a3}$, and $S_{a4}$ are the drive signals of four switching devices $V_{a1}$, $V_{a2}$, $V_{a3}$, and $V_{a4}$, respectively. $S_{a1}$ and $S_{a3}$, as well as $S_{a2}$ and $S_{a4}$, form two sets of complementary signals, which prevent the occurrence of a direct short circuit across the bridge arm. The operational state of the phase A bridge is $S_{a}$ and can be defined as one of three states: 1, 0, or −1. The corresponding output phase voltage $u_{ao}$ is the output from the points P, O, and N with the three voltage levels $U_{C1}$, 0, and $-U_{C2}$, respectively. Therefore, the switching condition and output voltage of the T-type three-level converter can be given in Table 1.

The combination where $S_{x1}=1$ and $S_{x2}=0$ is not listed as it represents a transitional or invalid state in standard SVPWM modulation strategies, which ensures continuous commutation paths and safety. To guarantee the periodic symmetry of the voltage output, it is essential to maintain $U_{C1}=U_{C2}=U_{dc}/2$, and the switching state of the T-shaped bridge can be expressed as

(1) $$S_{x}=\left\{\begin{array}{cc} 1 & \mathrm{if}\;S_{x1}=1,S_{x2}=1\\ 0 & \mathrm{if}\;S_{x2}=1,S_{x3}=1\\ -1 & \mathrm{if}\;S_{x3}=1,S_{x4}=1 \end{array}\right.$$

where x=a,b,c.

**Figure 2:**
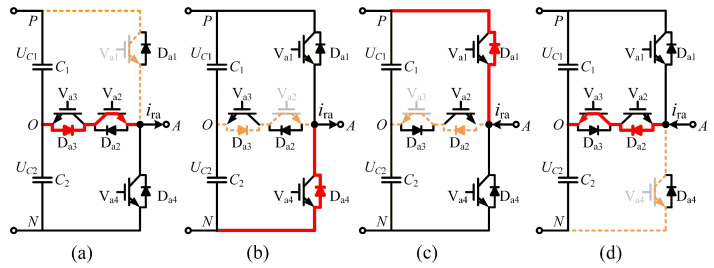
Impact on current paths with open circuit fault in (**a**) $V_{a1}$, (**b**) $V_{a2}$, (**c**) $V_{a3}$, and (**d**) $V_{a4}$.

Open-circuit failures of IGBTs generally arise from device fracture, disconnected bonding wire, loss of driving signal, and other reasons [28,29]. Figure 2 shows the impact of an open-circuit fault on the current paths of the T-type three-level converter, where the dashed line in yellow represents the current trajectory during normal operation and the bold solid line in red represents the current trajectory under abnormal operation with an open-circuit fault.

During the IGBT open-circuit fault condition, the output of the inverter no longer meets the requirements of Table 1, and the output phase voltage changes from $u_{ao}$ to $u_{ao\_f}$, as shown in Table 2. The change in the inverter output voltage further leads to distortion of the output current waveform. Here, $S_{a}$ denotes the condition of the bridge in phase A, $\mathrm{sign}(i_{ra})$ indicates the orientation of phase current, $u_{ao}$ represents the phase voltage under normal conditions, and $u_{ao\_f}$ is the phase voltage in the fault state.

**Figure 3:**
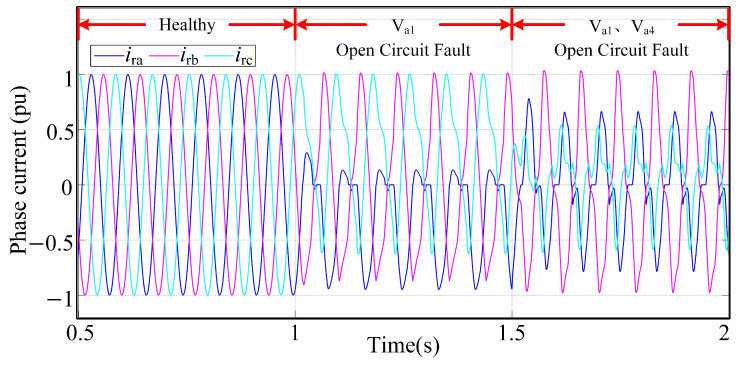
Three-phase currents of T-type three-level converter.

At the healthy state, the converter operates with symmetrical sinusoidal three-phase current signals. However, when a fault arises in the machine-side converter, the waveforms of the three-phase currents $i_{ra}$, $i_{rb}$, and $i_{rc}$ exhibit distortion, influencing the standard operation of the system. The degree of distortion to the three-phase currents obtained from simulation varies according to the type of fault, as shown in Figure 3.

## 3. Fault Detection Strategy

The data-driven methods possess the capability to extract the fault feature automatically from the historical data. However, the data-driven methods have the problems of high model construction cost, insufficient training data, and poor robustness and stability of the diagnostic model, which pose difficulties for practical applications. In this section, a simulation data transfer learning-based strategy for a T-type three-level power converter is introduced to tackle these issues.

### 3.1. Traditional Transfer Learning

**Figure 4:**
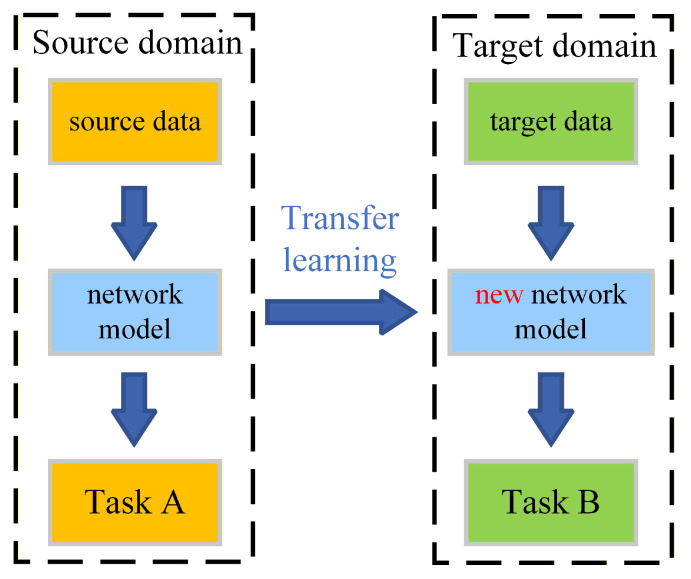
Basic principle of data transfer learning.

The basic principle of the transfer learning (TL) is shown in Figure 4. The TL relies on the existing network model that realizes task A on the source domain data, and a new network model is obtained by simple adjustment, which then realizes task B on the similar target domain data, thus avoiding the need to train the network model from scratch, and speeding up the training procedure of the network model that realizes the new task.

According to the different adjustment methods, the TL can be classified into feature-based transfer, relation-based transfer, and shared parameter-based transfer. Different TL methods are suitable for different application scenarios.

### 3.2. Simulation Data Transfer Learning

**Figure 5:**
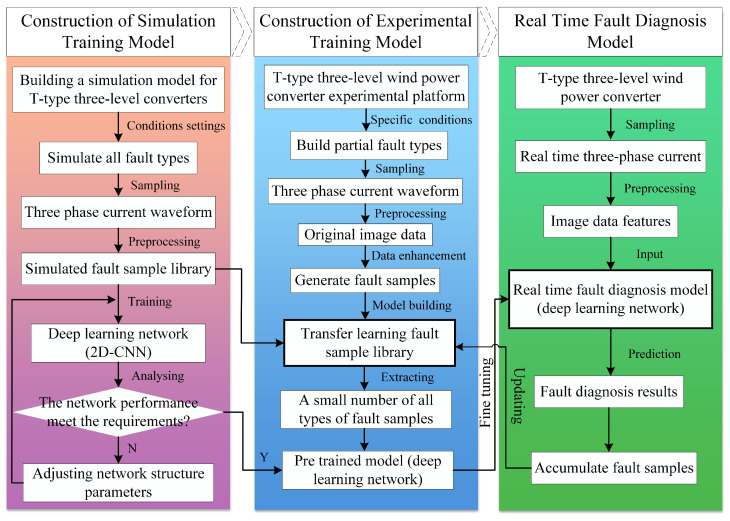
Overall framework of fault detection strategy.

With the fast development of the computer modeling technology, the simulation data based on the mathematical model of the power inverters has great similarity with the actual operation data, including the operation under healthy and faulty states. Therefore, the same feature extraction strategy could be adopted for the simulation and actual operation data, and the transfer method based on the shared parameters can be used to achieve the data transfer from simulation to reality for fault diagnosis. The overall framework of the fault detection strategy is shown in Figure 5, where the tasks are mainly divided into three blocks, namely, construction of simulation training model, construction of experimental training model, and real-time fault diagnosis model.

The overall framework of the fault detection strategy is shown in Figure 5, where the tasks are mainly divided into three blocks, namely, construction of simulation training model, construction of experimental training model, and real-time fault detection model.

1. Construction of simulation training model: The simulation model of a T-type three-level converter is first constructed, and the simulation fault sample library is constructed by simulating different faulty cases. The simulation fault sample library is used to train and optimize the network structure and parameters till the pre-trained model meets the requirement.
2. Construction of experimental training model: A small number of fault samples are obtained on a T-type three-level power converter experimental platform under specific operating states. The experimental and simulation fault samples are used to build a transfer learning fault sample library. A few fault samples are extracted from the transfer learning fault sample library to fine-tune the parameters of the pre-trained model, achieving transfer learning from simulation to experiment.
3. Real-time fault diagnosis model: In actual operation, the three-phase currents of a T-type three-level converter are sampled in real time, and the image data features are extracted to input into the real-time fault diagnosis model directly. Then the fault diagnosis results could be obtained without the manual processing of fault feature extraction, achieving end-to-end fault diagnosis.

## 4. Construction of Fault Detection Model

In this section, the construction of a fault diagnosis model according to the framework shown in Figure 5 will be discussed.

### 4.1. Simulation Environment and Data Generation

To validate the proposed strategy under challenging conditions, a simulation model of the T-type three-level converter is built in MATLAB/Simulink R2024a using standard ideal components. The simulation parameters are set to match the nominal ratings of the experimental platform. Crucially, the simulation intentionally employs ideal switches and voltage sources, ignoring complex non-linearities such as parasitic inductance, semiconductor voltage drops, and sensor noise.

This setup is chosen to demonstrate the calibration capability of the proposed method. In practical industrial scenarios, establishing a high-fidelity digital twin that perfectly matches all physical parasitic parameters is often computationally expensive and time-consuming. Therefore, the fidelity gap between this idealized simulation and the real experiment serves as the target domain shift. The proposed STLNet employs the fine-tuning stage of transfer learning to compensate for these unmodeled factors. This process effectively functions as a data-driven calibration, enabling robust diagnosis without relying on an intricate physics-of-failure model.

### 4.2. Data Preprocessing

In actual operation, the three-phase currents sampled in real time are time-series data, which cannot be directly input into 2D-CNN. Therefore, it is necessary to convert the time-series data into two-dimensional image data. Additionally, the amplitude, frequency, and phase of the three-phase current are influenced by working conditions and sampling moments. As a result, the waveform shapes of the three-phase current under different conditions with constant frequency time-domain sampling can vary significantly, making it difficult to directly use 2D-CNN to extract common features from the image data. Hence, data preprocessing is required to minimize the impact of operating states on the images, thereby obtaining high-quality image data samples.

The data preprocessing process converts the raw three-phase current data sampled during actual operation into high-quality image data samples, and can mitigate the influence of environmental factors on the three-phase current such as electromagnetic interference, vibration, and temperature. This is essential for building a fault sample library and training the fault detection model. The raw three-phase current obtained from sampling undergoes following preprocessing steps.

#### 4.2.1. Sliding Window Corner Domain Resampling

The sliding window is used to capture current waveforms with different initial phases, aiming to eliminate the impact of the initial phase on fault diagnosis results. Set the sliding window size to n=500 (corresponding to one cycle of current in angular resampling) and the sliding step size to τ=10, the three-phase current in one cycle will be divided into 50 vectors with different initial phases. Then it has

(2) $$I_{x}^{\left(k\right)}=\left[i_{x}\left(k\tau +1\right),i_{x}\left(k\tau +2\right),\ldots ,i_{x}\left(k\tau +L\right)\right]$$

where $I_{x}^{\left(k\right)}$ represents the *k*-th sample segment for phase *x*, with x∈{a,b,c} corresponding to phases A, B, and C, respectively. Here, *L* denotes the window length, τ is the sliding step size, and the elements within the vector represent the discrete current values sampled at equal angular intervals.

#### 4.2.2. Wavelet Packet Denoising

The collected three-phase current signals often contain high-frequency electromagnetic interference. To mitigate this, Wavelet Packet Decomposition is employed. The signals are decomposed into multiple frequency bands using a specific wavelet basis. Since noise typically manifests as high-frequency components with small amplitudes, a soft thresholding function is applied to the detail coefficients. Finally, the signal is reconstructed from the processed coefficients, effectively suppressing noise while retaining fundamental fault signatures [30].

#### 4.2.3. Hampel Filtering

**Figure 6:**
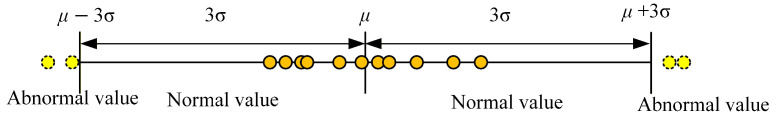
Hampel filtering principle.

Hampel filtering is used to eliminate abnormal burr in sampling three-phase current [31]. Figure 6 shows the principle of Hampel filtering, where a fixed-length window is used to segment sequential data, and the median value μ of this segment and the standard deviation σ of the data are calculated. Then the outliers falling outside the range [μ−3σ,μ+3σ] are considered anomalies and are replaced by μ.

#### 4.2.4. Amplitude Normalization

To eliminate the impact of load variations on the characteristics of three-phase currents, amplitude normalization of the three-phase currents is performed as follows:

(3) $$i_{x}^{\prime }=\frac{i_{x}-\min\left(i_{x}\right)}{\max\left(i_{x}\right)-\min\left(i_{x}\right)}$$

where x=a,b,c corresponds to phases A, B, and C, respectively. $\max(i_{x})$ and $\min(i_{x})$ represent the maximum and minimum values of the three-phase currents, respectively. The normalized current value ranges from 0 to 1.

#### 4.2.5. 2D Image Sample Generation

The three-phase currents are arranged in sequence to reflect their interrelationships and form a normalized two-dimensional matrix that represents the fault information of the converter. It has

(4) $$M=[I_{a};I_{b};I_{c}]$$

**Figure 7:**
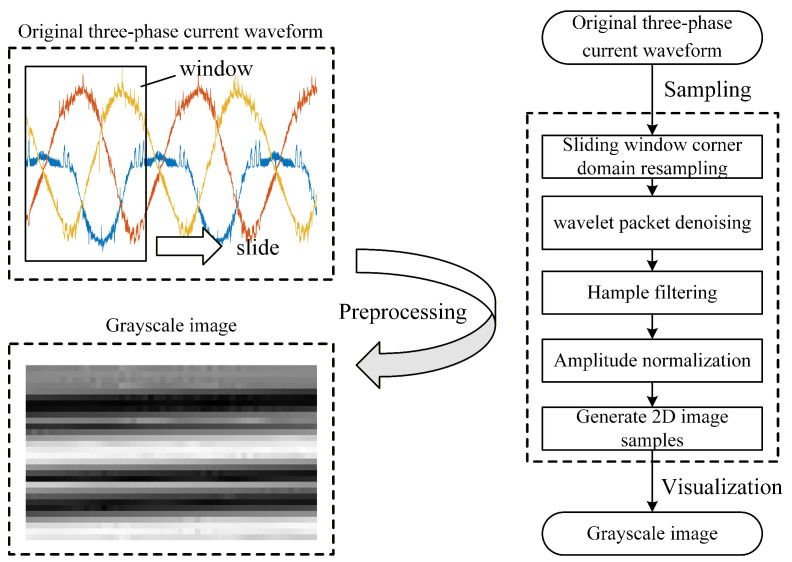
Data preprocessing process.

The obtained normalized two-dimensional matrix has element value ranging from [0, 1]. By converting these elements to a grayscale value in the range [0, 255], the samples can be visualized as a grayscale image. It is worth noting that, compared with time–frequency representations such as Short-Time Fourier Transform (STFT) or Wavelet Transform, the proposed 2D image generation method avoids complex signal transformation computations. This maintains a low computational overhead, which is crucial for the lightweight design objectives of this study. The data preprocessing process is shown in Figure 7.

**Figure 8:**
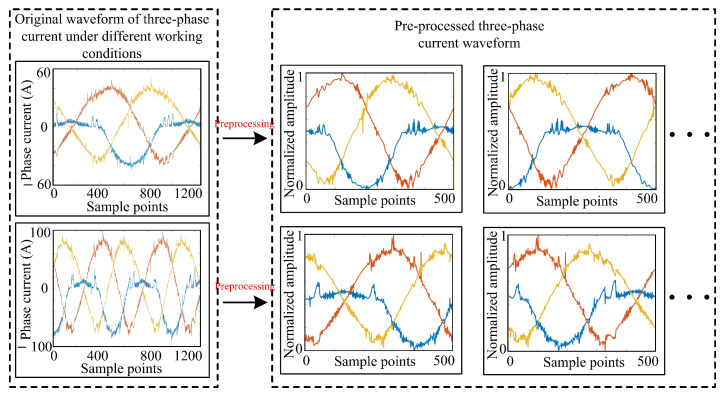
Comparisons of three-phase current waveforms with and without preprocessing.

Figure 8 shows comparisons of the three-phase current waveforms with and without preprocessing under the same type of fault and different operating states.

The raw three-phase current waveforms under different operating conditions exhibit significant differences in amplitude and number of cycles. However, after data preprocessing, the waveforms are transformed into single-cycle and unit amplitude signals without local detail features after denoising and filtering treatment. The preprocessing greatly enhances the similarity of different samples under the same fault type, which facilitates the extraction of common features by 2D-CNN. It is important to emphasize that the preprocessing steps constitute a coupled pipeline essential for bridging the gap between simulation and experimental domains. By aligning time scales under variable speeds, mitigating the SNR difference caused by sensor noise, and unifying signal amplitudes across different loads, these steps work synergistically to ensure feature consistency. Consequently, omitting any single step would degrade the quality of the input features, thereby hindering the effectiveness of the subsequent transfer learning process.

### 4.3. Data Enhancement

Data enhancement can increase the number of data samples and avoid the overfitting of the network. It is necessary to select appropriate data enhancement methods according to the specific characteristics of fault samples. According to the symmetry of three-phase currents, data enhancement methods including vertical flipping and current order alteration are proposed in this paper, which allow for the generation of sufficient training samples from a small number of collected fault samples, thereby making full use of the limited available fault samples.

**Figure 9:**
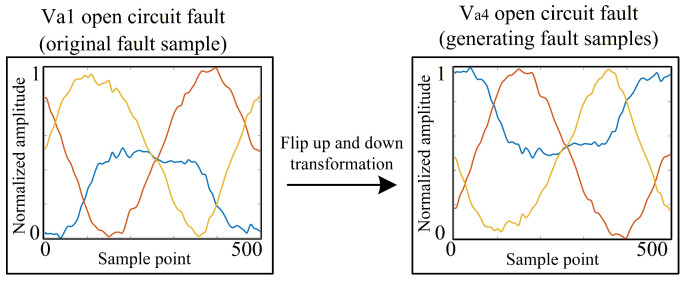
Vertical flipping transformation.

Vertical flipping involves rotating the three-phase current waveforms 180° around the time axis, thereby transforming samples of different fault types. Figure 9 shows an example of vertical flipping transformation for $V_{a1}$ open-circuit fault to $V_{a4}$ open-circuit fault.

The transformation can be expressed as

(5) $$I_{aug}^{\prime }=-I_{raw}$$

where $I_{raw}$ denotes the original raw current sample, and $I_{aug}^{\prime }$ represents the augmented sample obtained by negating the current amplitude. This operation, referred to as amplitude inversion, visually corresponds to reflecting the waveform across the horizontal time axis. Physically, it leverages the half-wave symmetry of AC signals to generate valid synthetic fault samples with reversed polarity.

The samples of single-device open-circuit fault and double-switch open-circuit fault in a single phase for a T-type three-level converter satisfied the vertical flipping transformation, as shown in Table 3, where x=a,b,c. The transformations shown in Table 3 are reversible, meaning that the original faulty device and the transformed faulty device can be interchanged.

The two-device open-circuit faults in two phases also satisfy vertical flipping transformation. Taking phases AB as an example, the specific transformation relationship is shown in Table 4, where the fault samples with the same number satisfy the vertical flipping transformation, and all the transformations are reversible. Similarly, the fault samples for phases BC and CA have the same relationships.

**Figure 10:**
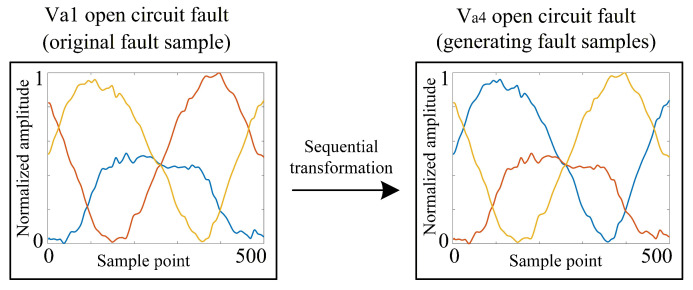
Example of current order alteration process.

Current order alteration involves rotating three phase currents in a specific order to convert fault samples between different types. An example of the current order alteration process for open-circuit fault from $V_{a1}$ to $V_{b1}$ is illustrated in Figure 10. The current order alteration process can be expressed with a normalized two-dimensional matrix, and it has the following:

(6) $$M_{\mathrm{new}}=\mathrm{Permute}\left(M_{\mathrm{old}}\right)$$

Table 5 shows the specific current order alteration relationships of the T-type three-level converter, where both the single-phase and two-phase fault samples satisfy this alteration. It is important to emphasize that these transformations are rigorously based on the inherent electrical symmetry of the three-phase topology. Consequently, the augmented samples maintain high statistical consistency with real-world fault scenarios. This physics-informed approach ensures that the model learns the fundamental fault mechanisms rather than fitting to artificial augmentation rules.

### 4.4. Construction of the Proposed STLNet Network Model

To improve the efficiency of building a fault detection model, a lightweight, highly generalized pre-trained model is designed and trained specifically for a T-type three-level converter. Based on this pre-trained model, DTL is used to develop a fault detection model.

First, different working conditions are set, and all fault scenarios are simulated in Matlab/Simulink. A set of fault samples for the T-type three-level converter is generated, which facilitates the construction of robust fault sample library. In the simulation model of the T-type three-level converter, four resistive loads under 10 Ω, 15 Ω, 20 Ω, and 25 Ω and five DC-link voltage $U_{dc}$ under 160 V, 170 V, 180 V, 190 V, and 200 V are set. The output frequency $f_{1}$ is set at 40 Hz and 50 Hz. Then, in total, 40 different conditions are adopted with varied DC-link voltage, load resistance, and output frequency.

Under different conditions, 79 operating states, including health and fault states, are simulated as shown in Table 6. Besides the health state, there are 12 types of single-device open-circuit faults, 18 types of single-phase double-device open-circuit faults, and 48 types of two-phase two-device open-circuit faults. For each state, three-phase current waveform in one cycle is captured and 50 image samples are generated through data preprocessing and data augmentation. Consequently, a total of 158,000 two-dimensional image samples are obtained for 40 working conditions.

Second, conduct experiments on the wind power converter experimental platform to create fault samples for specific types of faults. Preprocess these data to obtain two-dimensional image samples that can be directly input into 2D-CNN. Then, apply data augmentation methods to generate fault samples for all types of faults. This process enables the construction of a transfer learning fault sample library for the T-type three-level power converter.

In this paper, the structural parameters of the STLNet network designed for fault detection of a T-type three-level converter are shown in Table 7. The STLNet consists of 4 convolutional layers, 3 max-pooling layers, 1 global average pooling layer, 1 dropout layer, and 1 fully connected layer. Compared with pre-trained networks with millions of parameters based on the ImageNet dataset, the STLNet has fewer parameters and a simpler, more lightweight structure, making it more suitable for practical applications.

The network structure of the STLNet can be viewed as comprising two parts: a shallow feature extractor and a deep feature classifier. The shallow feature extractor is responsible for extracting fault features from the three-phase currents of the converter, while the deep feature classifier uses these features for fault classification. This paper employs a “shared parameters” transfer learning approach, using simulation data to train the feature extractor and achieve knowledge transfer for fault detection from simulation to reality.

In the first step, a fault detection model for the simulation source domain is trained using simulation fault samples, resulting in the STLNet pre-trained model. In the second step, the shallow feature extractor of the STLNet pre-trained model is frozen to share its parameters, and transfer learning samples are used to retrain and fine-tune the deep feature classifier. Specifically, the weights of all convolutional and pooling layers are locked (non-trainable). Only the parameters of the final fully connected layer are updated using the Adam optimizer with a reduced learning rate of $1\times 10^{-4}$ to ensure stable convergence. The fine-tuning process is conducted for up to 500 epochs with a batch size of 32, employing an Early Stopping mechanism to prevent overfitting. To verify robustness, the transfer experiment was repeated 10 times with random data sampling, yielding an average accuracy variance of less than 0.5%.

**Figure 11:**
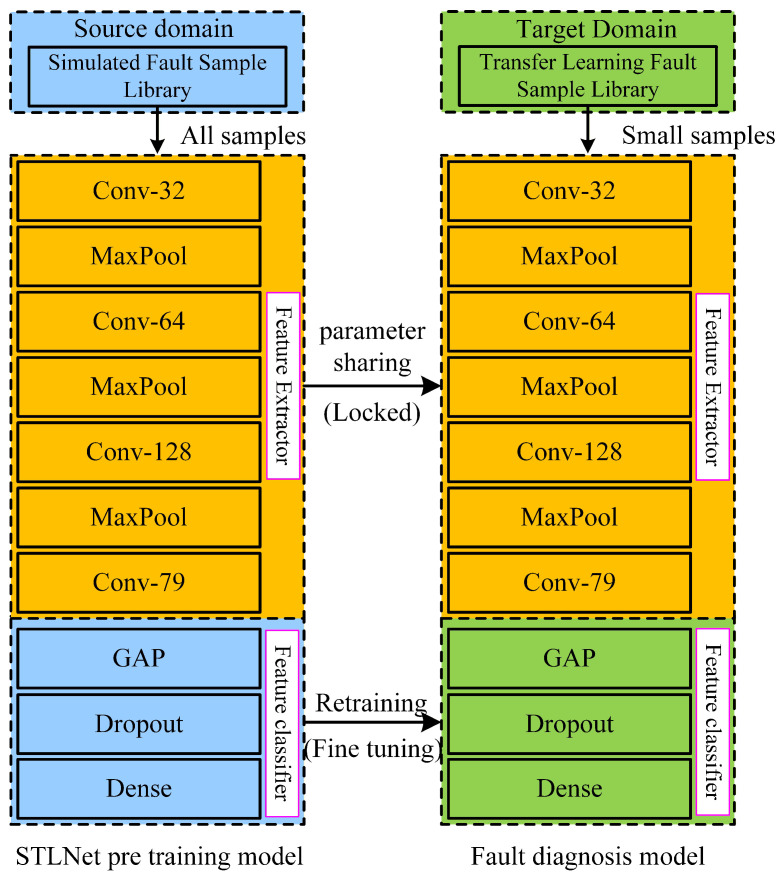
Schematic diagram of transfer learning for the fault detection model.

It can be seen from Figure 11 that only 6320 parameters of the fully connected layer (Dense) in the feature classifier need to be trained during the transfer learning retraining process. This significantly reduces the number of samples required for transfer learning, thereby greatly lowering the dependence on historical data and computational resources for constructing the converter fault detection model. Consequently, the cost of building the converter fault detection model is substantially reduced.

## 5. Experimental Verification

### 5.1. Experimental Setup and Data Acquisition

**Figure 12:**
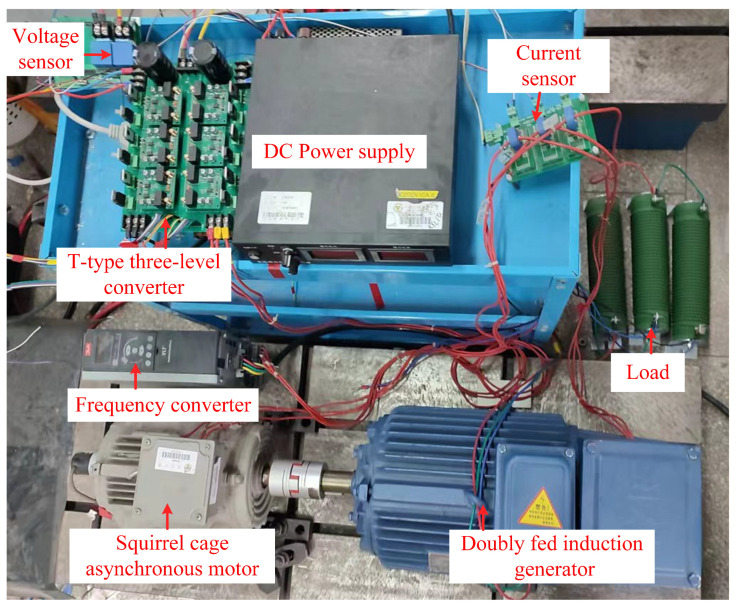
Experimental platform.

To validate the effectiveness of the proposed fault detection strategy derived from simulation data, an experimental setup of a T-type three-level converter is established in the laboratory, as shown in Figure 12. The IGBT open-circuit faults including a single-device open circuit fault, a single-phase two-device open circuit fault, and a two-phase two-device open circuit fault are analyzed in this section. The probability of more than two IGBT open-circuit faults is extremely low; therefore, they will not be discussed in this paper.

The experimental platform consists of a squirrel-cage induction motor, a motor driver, a DC power supply, a T-type three-level converter, a doubly fed induction generator (DFIG), two voltage sensors, three current sensors, and resistance load.

Within the experimental setup, the squirrel-cage induction motor is applied as the prime motor, and the motor driver provides the power supply for the induction motor. The T-type three-level converter is connected with the rotor windings of the DFIG shown in Figure 12, and the stator windings of the DFIG are directly coupled with the resistive load. The open-circuit fault of the IGBT is conducted by disconnecting the gate signal during the experiments, and all the tests are carried out under sub-synchronous state of the DFIG. The sampling frequency of the control system and data acquisition is set to 10 kHz. The parameters and working conditions are given in Table 8, where various current references $i_{rq}^{*}$ and rotor speeds $\omega_{r}$ of the DFIG are preset.

Under these varying test conditions, only one experiment is conducted under the healthy state, and two experiments under a single-device fault in phase A, four experiments under a two-device fault in phase A, and eight experiments under a two-device fault in phases A and B are conducted. This results in a total of 15 different operating states.

**Figure 13:**
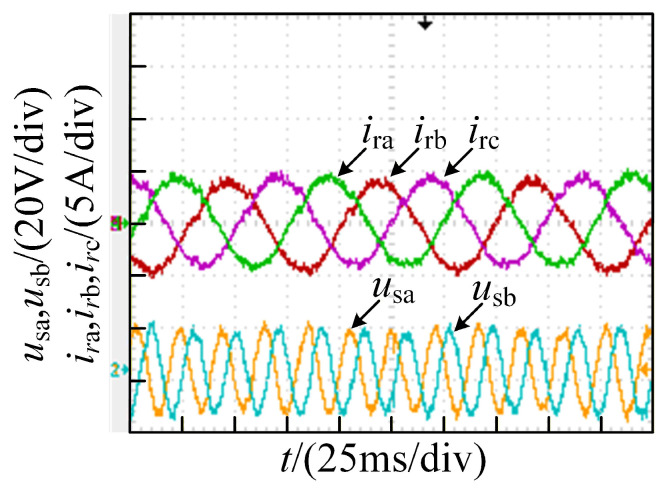
Three-phasecurrent waveforms under the healthy state.

**Figure 14:**
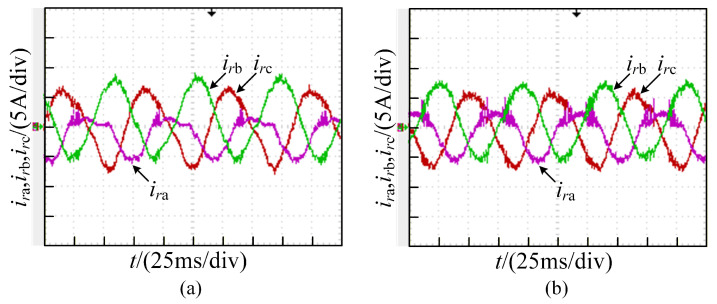
Currentwaveforms under single-device open circuit fault in phase A: (**a**) $V_{a1}$; (**b**) $V_{a2}$.

The current waveforms under the healthy state are illustrated in Figure 13. An open-circuit fault in phase A, as shown in Figure 14, causes distortions in all three-phase currents. When the outer device $V_{a1}$ experiences an open-circuit fault, the current distortions are relatively severe. In contrast, when the inner device $V_{a2}$ undergoes an open-circuit failure, the current distortions are comparatively mild, which agrees with the simulation results well.

**Figure 15:**
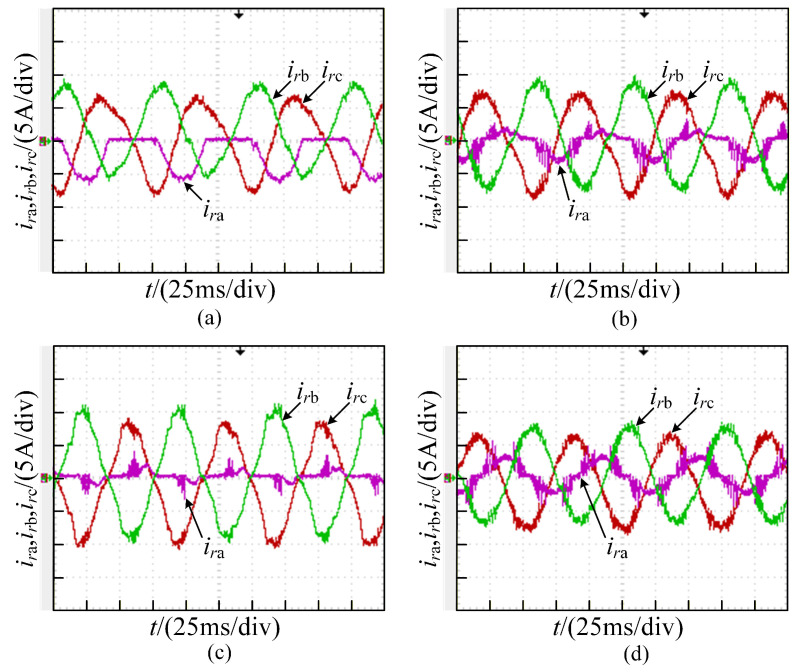
Current waveforms under two-device open circuit fault in phase A: (**a**) $V_{a1}$ and $V_{a2}$; (**b**) $V_{a1}$ and $V_{a3}$; (**c**) $V_{a1}$ and $V_{a4}$; (**d**) $V_{a2}$ and $V_{a3}$.

The two-device open-circuit fault in phase A, as shown in Figure 15, results in current distortions equivalent to the superposition of single-device open-circuit faults. The faulty phase exhibits more severe current distortions than the healthy phases.

**Figure 16:**
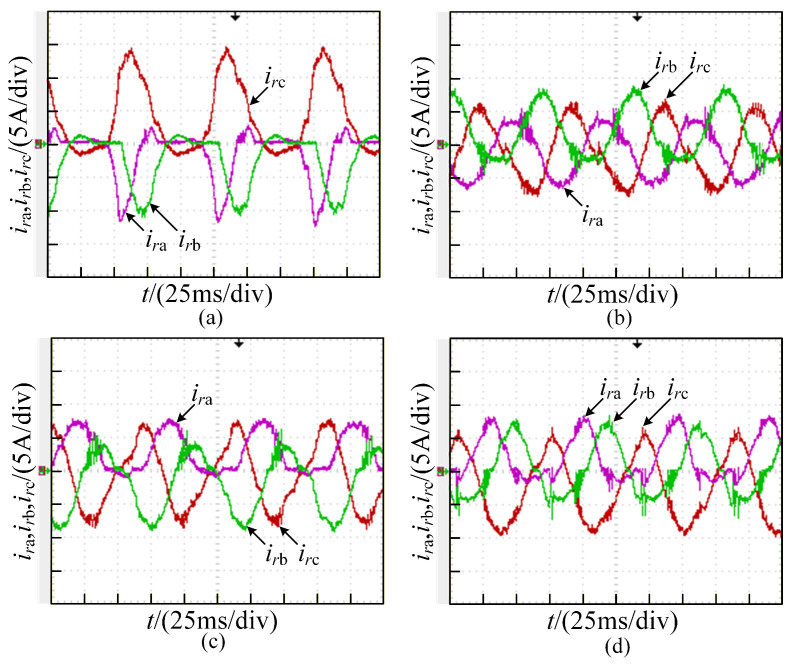
Current waveforms of two-device open circuit fault in phase AB: (**a**) $V_{a1}$ and $V_{b1}$; (**b**) $V_{a1}$ and $V_{b3}$; (**c**) $V_{a1}$ and $V_{b4}$; (**d**) $V_{a2}$ and $V_{b3}$.

The two-device open-circuit fault in phases A and B, as shown in Figure 16, results in current distortions equivalent to the superposition of single-device open-circuit faults in the faulty phases. This leads to severe three-phase current imbalance.

It can be seen from Figure 13, Figure 14, Figure 15 and Figure 16 that the degree of three-phase current distortions varies according to different fault locations. The rich fault characteristic information contained in these distorted current waveforms is the basis for training a deep learning network model. The open-circuit fault waveforms of the T-type three-level converter agree with the theoretical analysis and simulation results.

### 5.2. Experimental Results and Analysis

**Figure 17:**
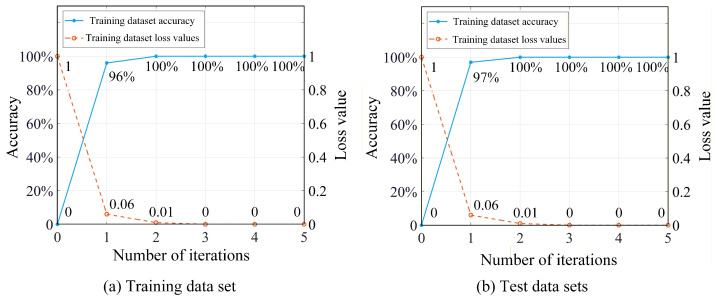
Pre-training results of STLNet.

A computer with CPU Intel Core i5@2.70 GHz, RAM 16.0 GB, and Windows 10 is adopted for constructing and training the model.During the pre-training of the STLNet, the number of training epochs is set to 5, with a batch size of 32. A dropout rate of 0.5 is applied to mitigate overfitting. The Adam optimizer is employed with an initial learning rate of 0.001, while categorical cross-entropy is adopted as the loss function. A total of 100,000 two-dimensional image samples from the 158,000 STLNet samples in the simulation fault sample library (approximately 60%) are randomly selected as the pre-training dataset, with the remaining 58,000 samples used as the test dataset. During the pre-training process, the accuracy and loss values of the STLNet for both the training dataset and the test dataset are shown in Figure 17.

As shown in Figure 17, after two iterations, the STLNet achieves 100% accuracy for both the training dataset and the test dataset. Additionally, the loss values for both datasets continue to decrease, indicating that the network has not yet overfitted and maintains good performance. Therefore, pre-training is stopped after two iterations, and the network structure and weight parameters are saved as the STLNet pre-trained model.

During the transfer learning retraining of the STLNet, the model is trained for 500 epochs with a batch size of 32. To enhance generalization, a dropout rate of 0.5 is applied. The optimization process is conducted using the Adam algorithm initialized with a learning rate of 0.001, and the training objective is defined by the categorical cross-entropy loss. In this process, the fault sample library for transfer learning is employed, where working conditions 1 and 4 are designated as the training dataset, while conditions 2, 3, and 5 are reserved for cross-condition testing. The training dataset consists of 7900 two-dimensional image samples collected under two conditions, including 79 operating states with 100 image samples in each operating state, which results in a balanced and representative dataset for model training.

**Figure 18:**
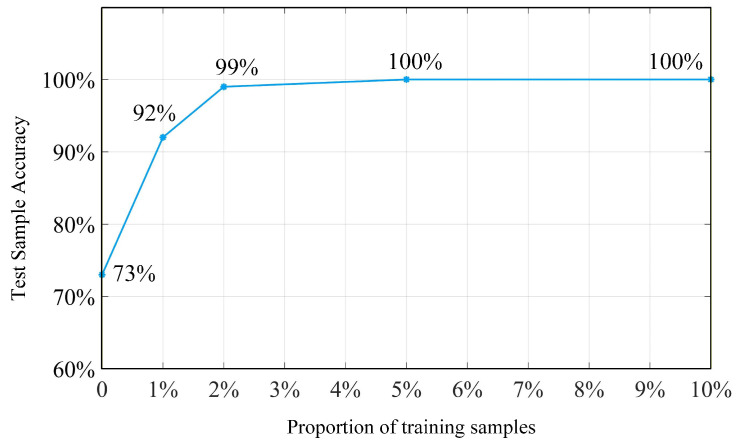
Transfer learning effects of training samples with different proportions.

Different proportions of samples are selected as training samples, and the accuracy of fault diagnosis after transfer learning is evaluated using the test samples, as shown in Figure 18. The accuracy of fault diagnosis using the pre-trained model directly is 73%. By retraining with only 5% of the training dataset samples, the network achieves a fault diagnosis accuracy of 100% on the remaining 95% of the samples, demonstrating the effectiveness of transfer learning.

**Figure 19:**
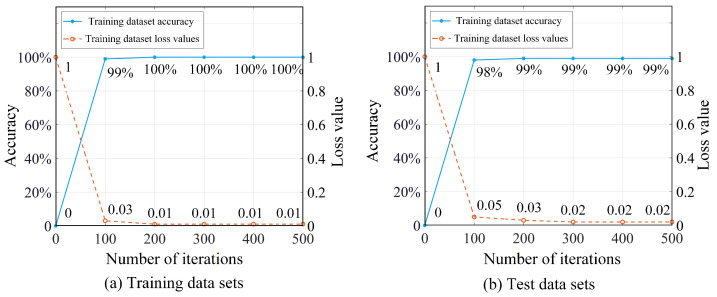
STLNet transfer learning training results.

A total of 5 samples from each of 100 two-dimensional image samples in normal and fault states (5% of the total) are randomly selected. Then a training dataset of 395 samples is constructed from 79 operating states, and the remaining 7505 samples are used as the test dataset. After 300 iterations during the transfer learning training process, the accuracy and loss value of the STLNet for both the training and test datasets are shown in Figure 19.

In Figure 19, the STLNet model has achieved an accuracy of 99% for both the training and test datasets after 200 iterations, and the loss value for both datasets continues to decrease. This indicates that the network has not yet overfitted and demonstrates excellent performance. Therefore, it is chosen to stop training after 200 iterations, save the network structure and weight parameters, thereby establishing the fault diagnosis model for the T-type three-level converter.

The proposed transfer learning STLNet is comparatively studied with the direct training STLNet and the classic LeNet-5 network, where the data of conditions 1 and 4 are used as the training dataset, and the data of conditions 2, 3, and 5 are used as the cross-condition test dataset. The cross-condition fault diagnosis results are presented in Table 9. Further analysis of the misclassification distribution reveals that the baseline models, namely, LeNet-5 and Direct-Training, most frequently confuse single-device open-circuit faults with same-phase dual-device faults. A typical example is distinguishing between a $V_{a1}$ fault and a combined $V_{a1}$ and $V_{a2}$ fault. These fault signatures are highly similar, especially under light-load conditions. In contrast, the proposed STLNet achieves high precision and recall, both exceeding 96%, across all fault categories, including single-device, dual-device, and dual-phase faults. This result demonstrates that the transfer learning strategy effectively captures the subtle discriminative features needed to separate these challenging fault pairs.

It can be seen from Table 9 that the transfer learning STLNet, compared with its direct training approach, provides more efficient feature extraction. It achieves higher fault diagnosis accuracy and exhibits stronger cross-condition adaptability with the same number of training samples, which saves time and computational resources during network training in practical applications. Additionally, STLNet shows superior generalization performance relative to the LeNet-5 network, achieving significantly higher diagnostic accuracy when both models are directly trained with only 5% of the samples.

**Figure 20:**
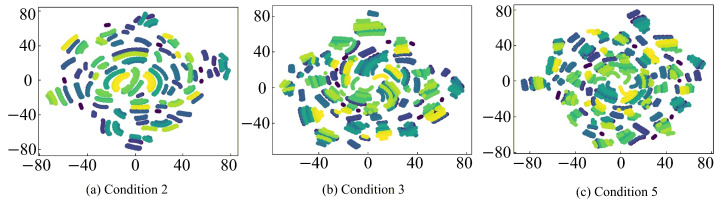
t-SNE visualization of input-layer features of 79 state types under operating conditions 2, 3, and 5.

**Figure 21:**
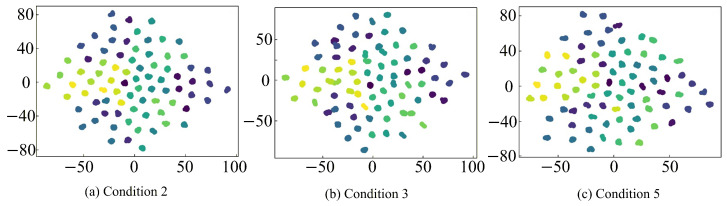
t-SNE visualization of classification-layer features obtained by transfer learning STLNet under working conditions 2, 3, and 5.

**Figure 22:**
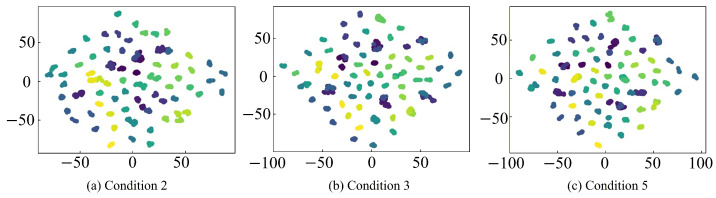
t-SNE visualization of classification-layer features obtained by direct training STLNet under working conditions 2, 3, and 5.

**Figure 23:**
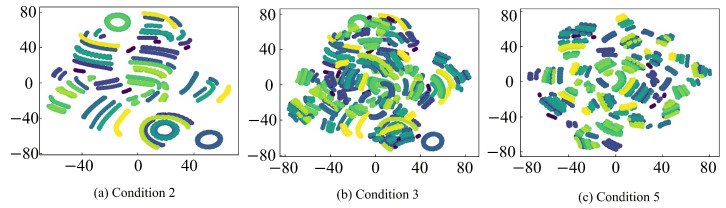
t-SNE visualization of classification-layer features obtained by LeNet-5 under working conditions 2, 3, and 5.

To further provide an intuitive analysis of the feature extraction capabilities of different fault diagnosis models, the t-SNE algorithm is employed to reduce the feature representations and turn them into a two-dimensional feature for visualization. The corresponding analysis results are shown in Figure 20, Figure 21, Figure 22 and Figure 23, where clusters with the same color and connectivity indicate the same type of normal or fault state.

It can be seen from Figure 20 that the input-layer feature distribution is highly chaotic and scattered, with substantial overlap and intermixing among different sample categories, which makes accurate discrimination extremely difficult. In contrast, as seen in Figure 21, Figure 22 and Figure 23, after feature extraction by the CNN within the fault diagnosis model, the feature distribution in the classification layer feature map is much clearer and concentrated, with distinct differences between different samples. This distinct clustering validates that the critical fault signatures are effectively preserved and aligned across domains by the STLNet, ensuring robust diagnosis without the need for complex time-frequency preprocessing. Furthermore, the tight grouping of simulated and experimental features in the latent space shown in Figure 22 and Figure 23 serves as visual evidence that the simulation-to-experiment domain gap, caused by parasitic parameters and non-ideal factors, has been effectively minimized by the transfer learning strategy.

Compared with the direct training STLNet and LeNet-5, the fault diagnosis model of the transfer learning STLNet demonstrates superior distinction in the classification layer feature maps. These feature maps exhibit minimal overlap, easily differentiating between different types of samples. This highlights the enhanced feature extraction capability of the fault diagnosis model. Consequently, the fault diagnosis model of transfer learning STLNet possesses robust cross-condition adaptability, offering substantial advantages in diagnostic accuracy and generalization capabilities.

## 6. Conclusions

This paper proposes a novel fault diagnosis strategy for T-type three-level power converters based on a lightweight simulation transfer learning network (STLNet). By integrating a physics-informed data augmentation strategy with a specialized 2D-CNN architecture, the proposed method effectively bridges the gap between abundant simulation data and scarce experimental fault data. Experimental validation on a wind power converter platform demonstrates the superiority of the proposed approach. Compared with direct training methods and the classic LeNet-5, the proposed strategy significantly reduces the dependency on real-world labeled data while maintaining high robustness against variable operating conditions. Furthermore, the lightweight design of the network ensures low computational complexity, making it suitable for practical deployment.

Despite these achievements, the current method still relies on a minimal amount of labeled experimental fault data for fine-tuning. In many industrial scenarios, acquiring even a small number of labeled fault samples can be challenging or impractical. Therefore, the dependence on real-world fault data remains a constraint for broader application.

Future work will prioritize realizing unsupervised transfer learning to eliminate the need for real-world fault data entirely. It is expected that high-performance fault diagnosis will be achieved using only simulation data and healthy experimental data by exploring advanced domain adaptation techniques. Additionally, while the experimental validation is primarily conducted under sub-synchronous operation, the employed angular resampling technique theoretically supports applicability to synchronous and super-synchronous regimes by normalizing the frequency dependence. However, explicit experimental verification under these specific operating modes remains to be performed in future studies.

## Figures and Tables

**Table 1:** Switching state and output voltage of T-Type three-level converter.

$S_{x}$	$S_{x1}$	$S_{x2}$	$S_{x3}$	$S_{x4}$	$u_{\mathrm{xo}}$
1	1	1	0	0	$U_{C1}$
0	0	1	1	0	0
−1	0	0	1	1	$-U_{C2}$

**Table 2:** Effect of open circuit fault on output voltage of T-type three-level inverter.

$S_{a}$	$\mathrm{Sign}\;(i_{\mathrm{ra}})$	Location	$u_{\mathrm{ao}}$	$u_{\mathrm{ao}\_f}$
1	1	$V_{a1}$	$U_{C1}$	0
0	1	$V_{a2}$	0	$-U_{C2}$
0	−1	$V_{a3}$	0	$U_{C1}$
−1	−1	$V_{a4}$	$-U_{C2}$	0

**Table 3:** Vertical flipping transformation of open-circuit fault samples in a single phase.

State	Faulty Device(s)					
**Original faulty device**	$V_{x1}$	$V_{x2}$	$V_{x1}V_{x2}$	$V_{x1}V_{x3}$	$V_{x1}V_{x4}$	$V_{x2}V_{x3}$
**Transformed faulty device**	$V_{x4}$	$V_{x3}$	$V_{x3}V_{x4}$	$V_{x2}V_{x4}$	$V_{x1}V_{x4}$	$V_{x2}V_{x3}$

**Table 4:** Vertical flipping transformation of fault samples in phases AB.

Faulty Device	$V_{a1}$	$V_{a2}$	$V_{a3}$	$V_{a4}$
$V_{b1}$	(1)	(2)	(3)	(4)
$V_{b2}$	(8)	(5)	(6)	(7)
$V_{b3}$	(7)	(6)	(5)	(8)
$V_{b4}$	(4)	(3)	(2)	(1)

**Table 5:** Current order alteration of fault samples in single-phase and two-phase faults.

Current Order	Original Fault					
	A	B	C	AB	BC	CA
abc	A	B	C	AB	BC	CA
bca	B	C	A	BC	CA	AB
cab	C	A	B	CA	AB	BC

**Table 6:** Total of 79 operating states of inverters for collecting simulation data.

State	Fault Location	Fault Type
Health	/	1
Single-device open circuit	$V_{a1},V_{a2},V_{a3},V_{a4},V_{b1},V_{b2},\cdots$	12
Single-phase two-device open circuit	$V_{a1}V_{a2},V_{a1}V_{a3},V_{a1}V_{a4},V_{a2}V_{a3},\cdots$	18
Two-phase two-device open circuit	$V_{a1}V_{b1},V_{a2}V_{b1},V_{a3}V_{b1},V_{a4}V_{b1},\cdots$	48

**Table 7:** Structural parameters of STLNet.

No.	Layer Name	Output Shape	Parameters
1	Convolutional Layer 1 (Conv-32)	(None, 3, 500, 32)	320
2	Max-Pooling Layer 1 (MaxPool)	(None, 3, 100, 32)	0
3	Convolutional Layer 2 (Conv-64)	(None, 3, 100, 64)	18,496
4	Max-Pooling Layer 2 (MaxPool)	(None, 3, 50, 64)	0
5	Convolutional Layer 3 (Conv-128)	(None, 3, 50, 128)	73,856
6	Max-Pooling Layer 3 (MaxPool)	(None, 3, 25, 128)	0
7	Convolutional Layer 4 (Conv-79)	(None, 3, 25, 79)	91,087
8	Global Average Pooling Layer (GAP)	(None, 79)	0
9	Dropout Layer (Dropout)	(None, 79)	0
10	Dense Layer (Dense)	(None, 79)	6320

**Table 8:** Experiment parameters and working conditions.

Parameter	Value	Parameter	Value
Rated power	2 kW	Rated current	6 A
Rated speed	750 rpm	Condition 3	$i_{rq}^{*}=6$ A, $\omega_{r}=660$ rpm
Condition 1	$i_{rq}^{*}=4$ A, $\omega_{r}=660$ rpm	Condition 4	$i_{rq}^{*}=6$ A, $\omega_{r}=720$ rpm
Condition 2	$i_{rq}^{*}=4$ A, $\omega_{r}=720$ rpm	Condition 5	$i_{rq}^{*}=5$ A, $\omega_{r}=690$ rpm

**Table 9:** Comparison of performance metrics for cross-condition fault diagnosis (mean ± standard deviation over 10 runs).

Condition	Model	Accuracy	Precision	Recall	F1-Score
2	STLNet	98.0±0.2%	98.2±0.2%	97.9±0.3%	98.0±0.2%
	DT S-CNN	92.0±0.6%	91.5±0.7%	92.3±0.6%	91.9±0.6%
	LeNet-5	47.0±1.5%	46.5±1.8%	48.2±1.6%	47.3±1.7%
3	STLNet	97.0±0.3%	97.5±0.2%	96.8±0.3%	97.1±0.2%
	DT S-CNN	91.0±0.5%	90.8±0.6%	91.2±0.5%	91.0±0.5%
	LeNet-5	49.0±1.3%	48.6±1.5%	49.5±1.4%	49.0±1.4%
5	STLNet	96.0±0.4%	96.4±0.3%	95.8±0.4%	96.1±0.3%
	DT S-CNN	91.0±0.7%	90.5±0.8%	91.4±0.7%	90.9±0.8%
	LeNet-5	47.0±1.6%	46.8±1.9%	47.5±1.7%	47.1±1.8%

## Data Availability

The data presented in this study are available on request from the corresponding author.

## References

[1] Cao T., Kong D., Hu C., Long B., Heldwein M.L. (2025). Exploring the Inherent Fault-Tolerance of Model-Free Predictive Control in Three-Level T-Type Converters. IEEE Trans. Power Electron..

[2] Long B., He Z., Garcia C., Rodríguez J., Chong K.T. (2024). A Model-Data Hybrid Driven Diagnosis Method for Open-Switch Faults in Three-Phase T-Type Grid-Connected Converters. IEEE J. Emerging Sel. Top. Power Electron..

[3] Li Z., Zhao B., Zhang X., Ma H. (2020). An IGBT open-circuit fault diagnosis method for grid-tied T-type three-level inverters. 2020 IEEE Energy Conversion Congress and Exposition (ECCE).

[4] Xu S., Zhang J., Hang J. (2017). Investigation of a fault-tolerant three-level T-type inverter system. IEEE Trans. Power Electron..

[5] Choudhary A., Fatima S., Panigrahi B.K. (2023). State of the art technologies in fault diagnosis of electric vehicles: A component-based review. IEEE Trans. Transp. Electrif..

[6] He J., Demerdash N.A.O., Weise N., Katebi R. (2017). A fast on-line diagnostic method for open-circuit switch faults in SiC-MOSFET-Based T-type multilevel inverters. IEEE Trans. Ind. Appl..

[7] Zhang W., He Y., Wang X., Chen J. (2023). A comprehensive method for online switch fault diagnosis and capacitor condition monitoring of three-level T-type inverters. IEEE Trans. Power Electron..

[8] Gou B., Xu Y., Xia Y., Deng Q., Ge X. (2020). An online data-driven method for simultaneous diagnosis of IGBT and current sensor fault of three-phase PWM inverter in induction motor drives. IEEE Trans. Power Electron..

[9] Choi S., Haque M.S., Tarek M.T.B., Mulpuri V., Duan Y., Das S., Garg V., Ionel D.M., Masrur M.A., Mirafzal B. (2018). Fault diagnosis techniques for permanent magnet AC machine and drives—A review of current state of the art. IEEE Trans. Transp. Electrif..

[10] Melluso F., Spirto M., Nicolella A., Malfi P., Tordela C., Cosenza C., Savino S., Niola V. (2026). Torque fault signal extraction in hybrid electric powertrains through a wavelet-supported processing of residuals. Mech. Syst. Signal Process..

[11] Chen J., Li T., He J., Liu T. (2025). An interpretable Wavelet Kolmogorov–Arnold Convolutional LSTM for spatial–temporal feature extraction and intelligent fault diagnosis. J. Dyn. Monit. Diagn..

[12] Huang W., Du J., Hua W., Lu W., Bi K., Zhu Y., Fan Q. (2021). Current-based open-circuit fault diagnosis for PMSM drives with model predictive control. IEEE Trans. Power Electron..

[13] Li Z., Wheeler P., Watson A., Costabeber A., Wang B., Ren Y., Bai Z., Ma H. (2020). A fast diagnosis method for both IGBT faults and current sensor faults in grid-tied three-phase inverters with two current sensors. IEEE Trans. Power Electron..

[14] Huang Z.J., Wang Z.S., Yao X.S., Zhang H.G. (2019). Multi-switches fault diagnosis based on small low-frequency data for voltage-source inverters of PMSM drives. IEEE Trans. Power Electron..

[15] Ge X., Pu J., Gou B., Liu Y.-C. (2018). An open-circuit fault diagnosis approach for single-phase three-level neutral-point-clamped converters. IEEE Trans. Power Electron..

[16] Liang Y., Wang R., Hu B. (2021). Single-switch open-circuit diagnosis method based on average voltage vector for three-level T-type inverter. IEEE Trans. Power Electron..

[17] Cai B., Zhao Y., Liu H., Xie M. (2017). A data-driven fault diagnosis methodology in three-phase inverters for PMSM drive systems. IEEE Trans. Power Electron..

[18] Xue Z.Y., Xiahou K.S., Li M.S., Ji T.Y., Wu Q.H. (2020). Diagnosis of multiple open-circuit switch faults based on long short-term memory network for DFIG-based wind turbine systems. IEEE J. Emerg. Sel. Top. Ind. Electron..

[19] Kim S.-H., Yoo D.-Y., An S.-W., Park Y.-S., Lee J.-W., Lee K.-B. (2020). Fault detection method using a convolution neural network for hybrid active neutral-point clamped inverters. IEEE Access.

[20] Yang J., Liu J., Xie J., Wang C., Ding T. (2021). Conditional GAN and 2-D CNN for bearing fault diagnosis with small samples. IEEE Trans. Instrum. Meas..

[21] Chen Z., Gryllias K., Li W. (2020). Intelligent fault diagnosis for rotary machinery using transferable convolutional neural network. IEEE Trans. Ind. Informat..

[22] Meng Z., Guo X., Pan Z., Sun D., Li S. (2019). Data segmentation and augmentation methods based on raw data using deep neural networks approach for rotating machinery fault diagnosis. IEEE Access.

[23] Guan X., Gao W., Peng H., Shu N., Gao D.W. (2022). Image-based incipient fault classification of electrical substation equipment by transfer learning of deep convolutional neural network. IEEE Can. J. Electr. Comput. Eng..

[24] Chen J., Zhang C., Chen A., Xing X. (2019). Fault-tolerant control strategies for T-type three-level inverters considering neutral-point voltage oscillations. IEEE Trans. Ind. Electron..

[25] Choi U.-M., Lee K.-B., Blaabjerg F. (2014). Diagnosis and tolerant strategy of an open-switch fault for T-type three-level inverter systems. IEEE Trans. Ind. Appl..

[26] Deng F., Zhu R., Liu D., Wang Y., Wang H., Chen Z., Cheng M. (2018). Protection scheme for modular multilevel converters under diode open-circuit faults. IEEE Trans. Power Electron..

[27] Katebi R., He J., Weise N. (2019). Investigation of fault-tolerant capabilities in an advanced three-level active T-type converter. IEEE J. Emerg. Sel. Top. Power Electron..

[28] Do D.-T., Nguyen M.-K., Quach T.-H., Tran V.-T., Blaabjerg F., Vilathgamuwa D.M. (2020). A PWM scheme for a fault-tolerant three-level quasi-switched boost T-type inverter. IEEE J. Emerg. Sel. Top. Power Electron..

[29] Ceballos S., Pou J., Zaragoza J., Robles E., Villate J.L., Martín J.L. (2011). Fault-tolerant neutral-point-clamped converter solutions based on including a fourth resonant leg. IEEE Trans. Ind. Electron..

[30] Zhang J., Sun W., Wang H. (2019). Fault Diagnosis of Wind Turbine Power Converter Considering Wavelet Transform, Feature Analysis, Judgment and BP Neural Network. IEEE Access.

[31] Pearson R.K. (2002). Outliers in process modeling and identification. IEEE Trans. Control Syst. Technol..

